# Correlations Between Root Metabolomics and Bacterial Community Structures in the *Phragmites australis* Under Acid Mine Drainage-Polluted Wetland Ecosystem

**DOI:** 10.1007/s00284-021-02748-7

**Published:** 2021-12-28

**Authors:** Chimdi M. Kalu, Henry J. O. Ogola, Ramganesh Selvarajan, Memory Tekere, Khayalethu Ntushelo

**Affiliations:** 1 Department of Agriculture and Animal Health, University of South Africa, Florida Science Campus, Roodepoort, 1709 South Africa; 2 Department of Environmental Science, University of South Africa, Florida Science Campus, Roodepoort, 1709 South Africa; 3 School of Agricultural and Food Sciences, Jaramogi Oginga Odinga University of Science and Technology, P.O Box 210-40601, Bondo, Kenya; 4 Laboratory of Extraterrestrial Ocean Systems (LEOS), Institute of Deep-Sea Science and Engineering, Chinese Academy of Sciences, No. 28, Luhuitou Road, Sanya, 572000 Hainan Province People’s Republic of China

## Abstract

Despite root microecology playing critical role in plant growth and fidelity, relatively few studies have focused on the link between the microbial communities and root metabolome in the aquatic macrophytes under heavy metal (HM) pollution. Using high-throughput metagenomic sequencing, targeted metabolomics and community-level physiological profile analyses, we investigated the symbiotic associations between *Phragmites australis* with rhizospheric bacterial communities under differing acid mine drainage (AMD) pollution. Results indicated that AMD pollution and root localization significantly affected root metabolome profiles. Higher accumulation of adenosine monophosphate, inosine, methionine, carnitine and dimethylglycine were observed in the rhizosphere under AMD than non-AMD habitat. Overall, the bacterial diversity and richness, and functional (metabolic) diversity were lower under high-AMD pollution. While non-AMD site was enriched with members of phylum Firmicutes, Proteobacteria were the most abundant taxa in the rhizosphere and endosphere under AMD-polluted sites. Further, plant growth promoting rhizobacteria (*Rhizobium*, *Delftia*, *Bradyrhizobium*, and* Mesorhizobium*) and metal-tolerant bacteria *(Bacillus*,* Arthrobacter*, *Massilia* and *Methylocystis*) were most abundant in AMD-polluted than non-AMD habitat. Finally, pH, TDS (total dissolved solids), Cu, Cr, Fe, and Zn content were the key environmental factors that strongly contributed to the spatial perturbation of rhizospheric metabolites, proteobacterial and acidobacterial taxa. Overall, the study linked the differential endospheric and rhizospheric bacterial community and metabolite profiles in *P*. *australis* under AMD environment and provided insights into HM adaptability and phytoremediation potential.

**Supplementary Information:**

The online version contains supplementary material available at 10.1007/s00284-021-02748-7.

## Introduction

*Phragmites australis* (Cav.) Trin. ex Steud., a common perennial emergent aquatic macrophytes, has emerged as one of the key species extensively applied in the natural and artificial wetland pollution control such as reclamation of acid mine drainage (AMD)-polluted and mine tailings wastewater [1]. There is extensive literature on *P*. *australis* interactions with the rich endophytic and epiphytic bacterial and fungal communities [2–4], much of which suggests that their symbiotic interactions play a key role in the plant fitness or invasion success under different environmental conditions [4].

Under AMD contaminated ecosystems, symbiotic interactions between *P*. *australis* rhizosphere with members of phyla Proteobacteria, Nitrospirae, Actinobacteria, Firmicutes, Acidobacteria, and Bacteroidetes predominates. Members of these phyla may include the beneficial plant growth promoting rhizobacteria (PGPRs) belonging to genus *Arthrobacter*, *Streptomyces*, *Mycobacterium* and *Devosia* [2, 5], metal-resistant aerobic microbes such as *Microbacterium hydrocarbonoxidans*, *Achromobacter xylosoxidans* and some species of *Bacillus* spp. and *Pseudomonas* spp. [6, 7], among others. PGPRs could greatly enhance the host plant growth, invasiveness, and sequestration of heavy metals (HM) through processes such as indole acetic acid (IAA) production, siderophore production, phosphate solubilization, and induced systemic resistance [8].

Interactions between plants and their associated microbiota also alters the production of both primary and secondary metabolites such as amino acids, organic acids, phenolic compounds, proteins and polysaccharides, that are important towards HM stress response. For example, significant increase in the levels of adenosine, adenine, guanine, lysine, leucine, glycine, alanine, arginine and jasmonic acid with concomitant decrease in glutamic acid and methionine in the roots of several plants under metal stress have been reported [9, 10]. Amino acids such as glycine, glutamine, serine, methionine, lysine, arginine, and proline are associated with HMs. Specifically, glycine is a precursor in the biosynthesis of glutathione (GSH), an important chelator of HM in cells, whereas proline play an important role in reducing free radicals formation and enhancing the level of GSH [11, 12]. In contrast, organic acids such as malate, citrate, and oxalate have been reported to be involved in the transportation of metals through xylem, and are involved in storage, transportation, sequestration and chelation of ions in vacuole and cytosols, and detoxification of HMs [9, 13]. For instance, the main organic acids that chelate HMs in plants include citric acid, malic acid, and oxalic acid [12, 14]. Despite the importance of plant-derived metabolites in the rhizosphere, relatively little is known about their spatiotemporal distribution and dynamics [15]. This is partly attributable to the fact that metabolomic study, particularly under natural systems, is still challenging as plant root exudation is a dynamic process involving diverse primary and secondary metabolites, with complex and cryptic signaling pathways. However, recent advances in disciplines of biological sciences such as metabolomics, transcriptomics, proteomics, ionomics etc., have started providing evidence that plants integrate information related to nutrient availability, external abiotic and biotic signals to plant–microbe interactions belowground via metabolites perturbations [16]. These interactions are key to their fitness or invasion success under different environmental conditions. Unfortunately, current understanding of the complex interactions between rhizosphere compartments, biogeography, the microbiome and its microbiome-reprogrammed systemic root exudation in *P*. *australis* under the multiple stress of low pH, sulfates and HM pollution, is still limited.

Under AMD-contaminated wetland ecosystem, spatially defined root exudation plays a key role in the rhizobiome assembly with the establishment of distinct fungal communities associated with specific root parts of *P*. *australis* [3]. Based on Baas-Becking hypothesis that “everything is everywhere, but the environment selects” [17], it is envisaged that deeper coverage of *P*. *australis* adaptation under AMD systems would further reveal pertinent bacterial community associations and adaptation to HM and nutrient-enriched rhizosphere. A better knowledge on the root rhizosphere continuum, metabolites–microbial community dynamics and metal interactions is also needed to develop robust and/or improved *P*. *australis*-based phytorhizoremediation of AMD-polluted environments.

In this study, an integrated targeted metabolomics and metagenomics was used to evaluate the differential primary metabolome exudation profiles and the concomitant bacterial community assemblages in the root system of *P*. *australis* under differing AMD pollution. Biolog Ecoplates™ based CLPP analysis was also used to infer microbial metabolic/functional diversity within and among rhizosphere soil communities in the different AMD polluted sites. The study aimed to answer the question, how does the root physiology including root exudates shape *P*. *australis* rhizomicrobiome assembly and functional diversity, its adaptability and HM sequestration capacity under AMD pollution?

## Materials and Methods

### Site Description and Sample Collection

Roots of *P*. *australis* plants, surrounding sediment soils and water were sampled from two mine tailing dams, Lancaster 3 (S26°07.820′, E27°46.680′) and Wuinze 17 (S26°07.171′, E27°43.305′), located in Gauteng province, South Africa. Based on HM pollution levels, Lancaster 3 and Wuinze 17 sites were grouped as high-AMD and mid-AMD polluted environments, respectively. Samples were also collected from Florida Lake (S26°10.625′, E027°54.220′), a reclaimed recreational freshwater dam in the same gold mining belt of Roodepoort, Gauteng, South Africa, as a non-AMD site. Summary of the typical characteristics including selected physicochemical parameters of the sampling sites is provided in Supplementary Table S1. Sampling and fractionation of the root system into rhizosphere and the root endosphere for metagenomic experiments were performed as described by Kalu et al. [3].

### Targeted Metabolomic Analyses

To extract primary metabolites, 10 g of fresh rhizosphere soil and root samples were ground in 1 ml 75%:25% v/v methanol:water solution using pestle and mortar. The mixture was transferred into a clean tube, vortexed briefly, then sonicated for 10 min before being centrifuged at 15,000 × *g* for 5 min at 4 °C. The supernatant (800 μl) was filtered through a 0.22-μm filter membrane syringe, before the filtrate was lyophilized using a freeze dryer, dissolved in water, and analyzed for key metabolites of central metabolic pathways, including organic acids, amino acids, nucleotides, vitamins and signalling molecules by UHPLC–MS/MS.

The UHPLC-MS*/*MS analyses were performed using a Nexera LC system (Shimadzu Corporation, Kyoto, Japan) coupled to a LCMS-8040 triple quadrupole mass spectrometer (Shimadzu Corporation, Kyoto, Japan) using Shim-pack Velox® PFPP (pentafluorophenylpropyl) column (150 × 2.1 mm, 3 μm; Shimadzu Corporation, Kyoto, Japan) and an octadecylsilylated silica column (InertSustain C18, 150 × 2.1 mm, 3 μm; GL Sciences, Tokyo, Japan) for cationic and anionic analyses modes, respectively, as described previously [3].

### Illumina 16S rDNA High-Throughput Sequencing

Faecal/Soil Total DNA™ extraction kit (Zymo Research Corporation, CA, USA) was used to extract environmental DNA from ground root endosphere and rhizospheric soils according to manufacturer’s instructions and stored at − 20 °C prior to further analysis. For each sample, DNA was extracted in triplicates and pooled together. 16S rRNA gene fragments libraries preparation using 27F and 518R primer pairs, fused with MiSeq adapters and heterogeneity spacers was done according to protocol described by Ogola et al. [18]. Resultant libraries were sequenced by paired end (300 bp reads) sequencing v.3 chemistry along with its multiplex sample identifiers on the Illumina MiSeq Platform according to standard protocol.

Raw Fastq files from Illumina sequencing have been deposited at the NCBI sequence read archive (SRA) as BioProject ID PRJNA742387. The sequences were curated using the mothur v1.40.5 pipeline implemented in Nephele (v2.2.8) [19]. Sequences were assigned to operational taxonomic units (OTUs) using a dissimilarity cutoff = 0.03 and classified to representative microbial taxa against the nonredundant SILVA v132 ribosomal RNA database.

### Catabolic Activity of Bacterial Communities

The metabolic fingerprint of rhizospheric soil bacterial communities referred to as community-level physiological profile (CLPP) was determined using Biolog Ecoplates™ system (Biolog Inc., Hayward, CA, USA) as described by Mendes et al. [20]. Absorbance was measured at 590 nm with a VarioSkan Flash (Thermo Fisher Scientific Corporation, Waltham, MA, USA) absorbance scanner at 0, 24, 48, 72, 96, and 120 h after incubation.

### Statistical Analyses

For metabolomic data, the corrected peak area values were subjected pareto-scaling normalization before multivariate statistical analysis by Principal Component Analysis (PCA) and heatmap analysis. A supervised differential metabolite abundance analysis was also performed using *ALDEx2* package [21]. *ALDEx2* uses the centred log-ratio (clr) transformation and Dirichlet Monte-Carlo instances to infer biological and sampling variation, calculating the expected Benjamini–Hochberg false discovery rate (FDR) based on a Wilcoxon Rank Sum test and Welch’s *t*-test, a Kruskal–Wallis test, a generalized linear model, or a correlation test.

The plot_richness function of *phyloseq* package was used to calculate alpha diversity indices such as *Chao1*, Shannon, Simpson, ACE for the bacterial community datasets as described by Ogola et al. [18]. The dominant OTUs at different taxonomic levels were used to generate stacked bar charts and heatmap using *ggplot2* and *heatmap.2* packages in R, respectively. To delineate the core microbiome in the environmental samples, the “amp_venn” function of *ampVis2* package were used. β-diversity based on non-metric multidimensional scaling (NMDS) ordination of Weighted UniFrac distance between samples of across AMD habitats was also performed using metaMDS function of the *vegan* package of R.

To analyze the CLPP data, negative OD values were initially adjusted to zero and absorbance values of 0.2 or higher were considered positive to reduce the number of false positives. Data was also adjusted according to the modified Gompertz equation [22]. Carbon-consumption kinetics such as average well colour development (AWCD), substrate average well colour development (SAWCD), substrate richness (SR), and Shannon diversity index (H′) were calculated as described previously [20].

## Results

### Metabolome Profile in *P*. *australis* Root Under AMD Pollution

**Fig. 1:**
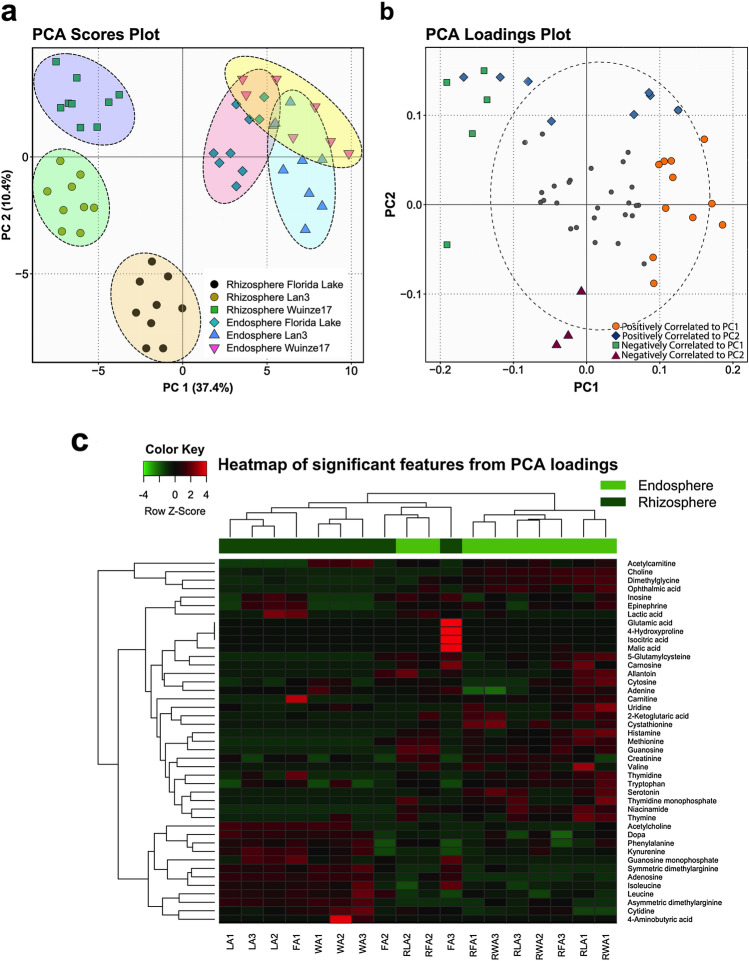
Principal component analysis of metabolic profiles in *P*. *australis* endosphere and rhizosphere under different AMD pollution gradient. **a** Score plot of PC2 versus PC1. Black ellipses represent the 90% confidence intervals for each group. **b** Loading plot of PC2 versus PC1 showing metabolites with significant loadings at *P* < 0.1. **c** Supervised hierarchical clustering heatmap of significant metabolites identified by PC loadings. The color scheme red and green indicate high and low concentrations of metabolites, respectively (Color figure online)

A total of 73 metabolites (18 and 55 features in anionic and cationic mode analyses, respectively) was identified based on the multiple reaction monitoring transitions and retention times of standard compounds (Supplementary Materials Table S2). These included amino acids, nucleotide/nucleosides, carbohydrates, organic acids, vitamins, and other known plant metabolites. PCA showed that rhizospheric samples were well separated from endosphere samples along PC1 axis, accounting for 37.4% variation (Fig. 1a). In contrast, no clear separation in the ordination space of endospheric samples was observed in terms AMD pollution gradient. Rhizospheric samples also exhibited a clear separation according to sampling sites. This suggests that the impact of AMD pollution on *P*. *australis* metabolite perturbations is more pronounced in the rhizospheric than endospheric root compartment.

PCA loadings analysis identified 43 potentially significant (*P* < 0.1) metabolites that exerted greater influence on separation of samples in the ordination space (Fig. 1b, Supplementary Material Table S3). Separation along the PC1 axis identified 18 features (metabolites) that showed high variation by root localization (rhizosphere *vs*. endosphere). This included methionine, ophthalmic acid, niacinamide, guanosine, choline, symmetric dimethylglycine and adenosine among others. In contrast, separation along the PC2 axis identified 14 features that showed high variation by sampling site (AMD pollution gradient) (Supplementary Materials Table S3). Heatmap showing both the hierarchical clustering analysis (HCA) and differential abundance of the 43 significant metabolites revealed overall clustering of samples in the ordination space according to root localization (Fig. 1c).

**Fig. 2:**
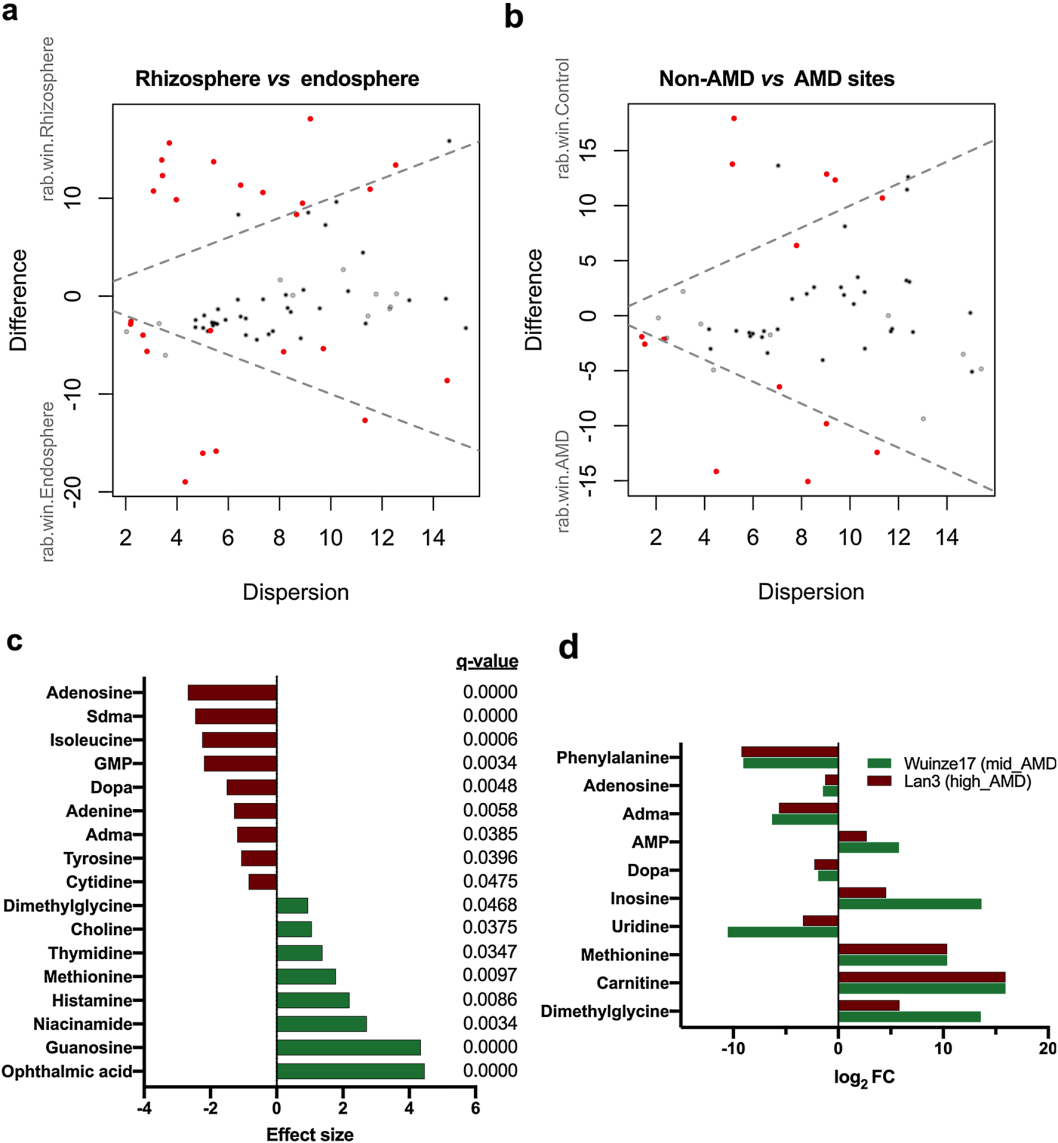
Relationship between metabolite abundance and observed differences in the primary metabolome of the *P*. *australis* rhizosphere and endosphere under AMD conditions (**a**, **b**). In both plots red represents differentially abundant features called with *q* < 0.05; grey are abundant, but not non-differentially abundant; black are rare, but not differentially abundant. **c** Plot showing the effect size and BH-adjusted *P* values (*q*-values) of the significant metabolites between rhizosphere (green) and endosphere samples (red). **d** Log2 Fold Change analysis of differentially abundant rhizospheric metabolites related to AMD pollution gradient using non-AMD site as reference. Sdma, symmetric dimethylglycine; GMP, Guanosine monophosphate; Adma, symmetric dimethylglycine; and AMP, Adenosine 3′,5′-cyclic monophosphate (Color figure online)

Differential analysis based on ALDEx2 analysis also identified 8 metabolites overrepresented in *P*. *australis* rhizosphere (Fig. 2a, c, Supplementary Material Table S4). In contrast, 9 metabolites (four nucleoside/nucleotides, two amino acids and their derivatives, and dopa) were found to be reduced in the rhizosphere samples but overrepresented in endosphere samples. To further identify metabolites significantly contributing to the separation of the rhizosphere according sampling site (Fig. 1a), samples were grouped into non-AMD and AMD sites (Mid- and High-AMD) before subjecting to ALDEx2 analysis. The effect size plot of non-AMD *vs*. AMD sites is presented in Fig. 2b. Notably, 5 metabolites each were significantly overrepresented in non-AMD and AMD site samples, respectively. Further decomposition of the samples into non-AMD, mid-AMD and high-AMD sites showed that adenosine monophosphate (AMP), inosine, methionine, carnitine and dimethylglycine accumulation was higher in both mid-AMD (log_2_FC = 5.8–15.9) and high-AMD (log_2_FC = 2.7–15.9) than in non-AMD environment (Fig. 2d). However, non-AMD *vs.* mid-AMD and non-AMD *vs.* high-AMD analyses revealed that uridine, dopa, asymmetric dimethylglycine, adenosine and phenylalanine accumulation was reduced under AMD polluted environment.

### Variation of *P*. *australis* Root Bacterial Community Under AMD Pollution

Overall, a total of 161,506 high-quality reads (5227 to 47,137) were obtained from all the six samples based on the 16S rRNA gene-based community analysis (Table 1). Good’s coverage across the samples was > 98%. Thus, the sampling depth was enough to estimate the diversity enclosing all major bacterial groups inhabiting the endosphere and rhizosphere under different AMD environments.

**Table 1:** Alpha diversity indices for soil rhizosphere and root endosphere bacterial communities associated with the roots of *Phragmites australis* growing in acid mine drainage (AMD) habitats

Indices^†^	Rhizosphere			Endosphere		
	Non-AMD	Mid-AMD	High-AMD	Non-AMD	Mid-AMD	High-AMD
Target reads	19,863	42,634	16,709	29,936	47,137	5227
Observed OTUs	620	1283	229	2146	853	457
ACE	1228	2355	244	2310	1009	483
*Chao1*	672	1110	223	2045	716	465
Shannon	2.38	2.87	0.86	4.75	2.99	3.97
Simpson	0.27	0.20	0.77	0.05	0.14	0.07
Good’s coverage (%)	98.6	99.3	99.6	99.4	99.8	98.9

^**†**^Diversity indices (observed OTUs, *Chao1*, Shannon, and Simpson) were based on rarefied datasets of 5227 sequences representing the lowest number of reads in a sample

### Changes in OTU Number, Richness, and Diversity of Bacterial Communities

The number of OTUs and alpha diversity indices are shown in Table 1. A total of 5588 OTUs (ranging between 229 and 2146) were obtained from all the six samples. Both species richness (OTU, *Chao1*, and ACE) and diversity estimates (Shannon and Simpson index) differed between the sampling sites showing a decreased trend under high-AMD habitat (Table 1). However, mid-AMD site had comparatively higher species richness and diversity of bacterial taxa than other habitats. Overall, the biodiversity indices separated the AMD habitat from the non-AMD site.

### Dynamics in Microbial Community Structure

**Fig. 3:**
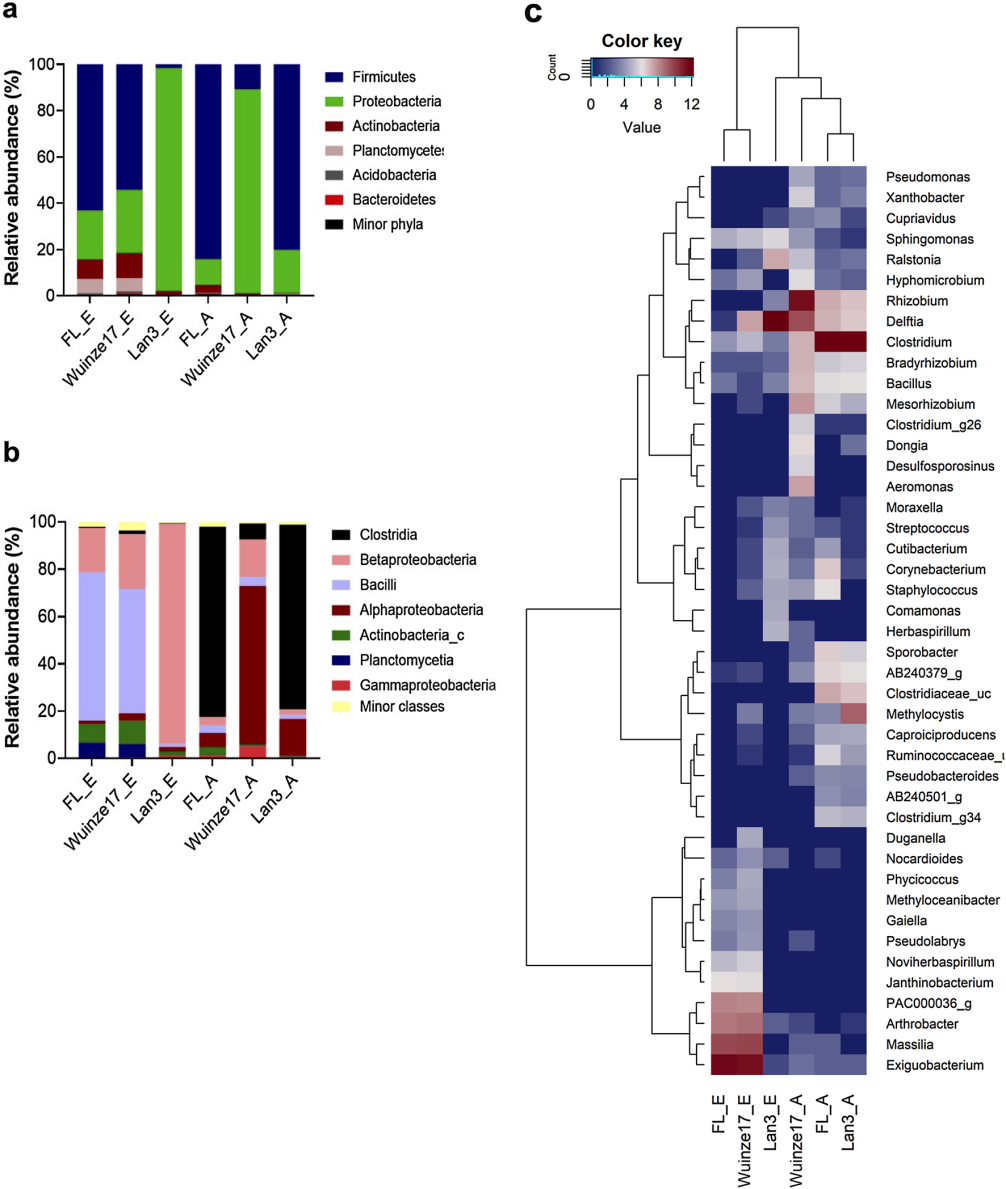
Stacked and heatmap plots of the relative abundances of major bacterial taxa. Stacked plots of major phyla (**a**) and classes (**b**) associated rhizosphere and the root endosphere of *Phragmites australis* growing in different AMD polluted environments. FL_E, Wuinze17_E and Lan3_E denotes the rhizospheric samples, while FL_A, Wuinze17_A, and Lan_A are endosphere samples of non-AMD, mid-AMD and high-AMD sites, respectively, and. **c** Heatmap of log_2_ normalized counts of the 40 most abundant genera. The heatmap color (blue to brown) represent the row z-score of the mean relative abundance from low to high (Color figure online)

A total of 30 phyla, 91 classes, 197 orders, 429 families and 1483 genera were identified in the bacterial community. Firmicutes, Proteobacteria, Actinobacteria, Planctomycetes, Acidobacteria and Bacteriodetes were the most abundant phyla (Fig. 3a), with discernible variations in the bacterial taxa according to root compartments and AMD habitat observed. The non-AMD site had higher abundance of phylum Firmicutes, accounting for 60% and 90% of taxa in rhizosphere and endosphere, respectively. In contrast, Proteobacteria constituted the major phylum in AMD sites accounting for ~ 90% abundance of taxa in rhizosphere and endosphere. Clostridia, β-proteobacteria, Bacilli, α-proteobacteria, Actinobacteria_c, Planctomycetia and γ-proteobacteria were the most abundant classes (Fig. 3b). Strikingly, β-proteobacteria was most abundant in high-AMD rhizosphere. However, high-AMD sites and non-AMD endosphere shared similar abundance of the class Clostridia, while α-proteobacteria were relatively more abundant in mid-AMD habitat.

The relative abundance at the genera level (Fig. 3c) showed variations across the three habitats and root compartments. Mid-AMD and non-AMD rhizosphere samples clustered together, with order Bacillales (genus *Exiguobacterium*), Burkholderiales (genus *Massilia*), Actinomycetales (genus *Arthrobacter*) and Planctomycetales (unclassified Planctomyceteae PAC000036) being the most abundant. In contrast, high-AMD and non-AMD endosphere samples clustered together, with genus *Clostridium* as the most abundant taxa. Interestingly, only high-AMD site endosphere exhibited higher abundance (9.92%) of the genus *Methylocystis*. In contrast, high abundance of genus *Rhizobium* (53.7%) and *Delftia* (14.5%) was detected in mid-AMD samples.

### Root Core Microbiome are Differentially Enriched According to AMD Pollution

**Fig. 4:**
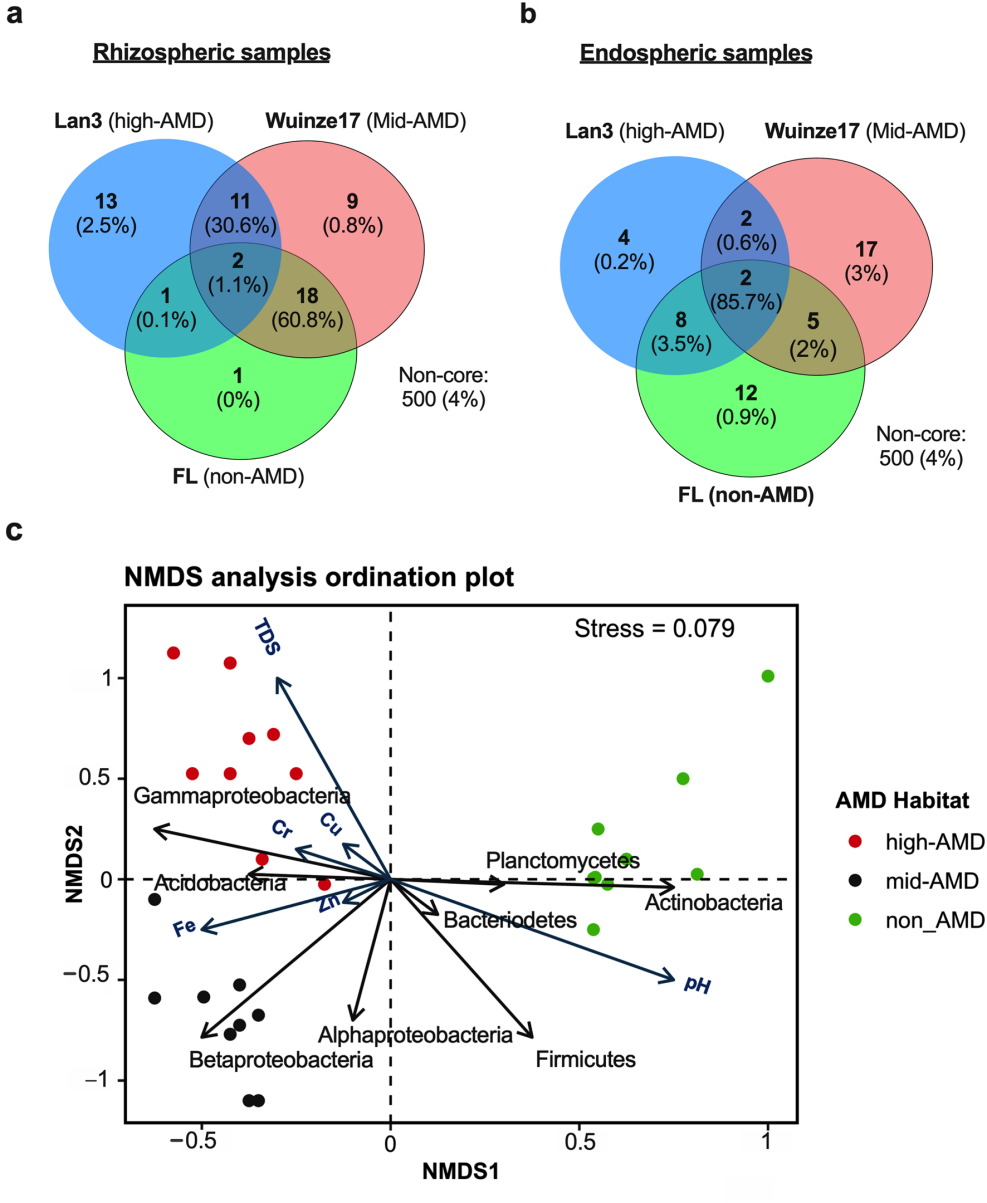
Venn diagrams of core microbiome and nonmetric multidimensional scaling (NMDS) analysis plot. Venn diagram showing number of unique and shared OTUs associated with rhizosphere **a** and root endosphere **b** of *Phragmites australis* under different AMD environments. **c** NMD plot derived from the Weighted Unifrac showing the association of bacterial communities and metabolites with the environmental variables: pH, TDS, Fe, Cr, Cu and Zn in the rhizosphere of *P*. *australis* under differing AMD pollution

A total of 42 and 60 OTUs (7.9 and 11% of OTUs in the rhizosphere and root endosphere, respectively) were obtained as the core microbiota, representing ~ 96 and 95.9% of the total reads generated for the rhizosphere and root endosphere, respectively (Fig. 4a, b). Interestingly, 13 OTUs assigned to Chromatiales *DQ378269*, and genus *Moraxella*, *Stenotrophomonas*,* Rhizobium*, *Aquamicrobium, Bradyrhizobium,* *Comamonas*, *Herbaspirillum*), *Corynebacterium*, *Cutibacterium*, *Staphylococcus* and *Streptococcus* were identified as unique taxa in high-AMD rhizosphere. In contrast, only 1 (unclassified Exiguobacteriaceae) and 13 OTUs were unique taxa in non-AMD and mid-AMD rhizosphere, respectively. In the endosphere, 12 OTUs representing 85.7% of the total reads were shared across the three habitats as core microbiome (Fig. 4b). These were mainly assigned to the genus *Clostridium*, *Delftia*, *Mesorhizobium*, *Methylocystis*, *Pseudomonas*, *Rhizobium*, *Sporobacter*, *Xanthobacter*, *Bacillus*, and *Bradyrhizobium*. Conversely, endosphere also had very low abundance (representing < 4.1% of the total reads generated for the endosphere) of unique taxa in the three habitats.

### Correlation Between AMD Physicochemical Indices, Rhizospheric Metabolites and Bacterial Community

NMDS ordination plot revealed significant variation in metabolite profile and bacterial community between rhizospheric samples across the AMD habitats (Fig. 4c). The NMDS stress of 0.079 confirmed that metabolite profile and bacterial community could be accurately described only in two dimensions. Vector fitting of the environmental parameters against the ordination plot of NMDS revealed that metabolite profile and bacterial composition discrimination on the first and second axis. This was mainly explained by pH (*r*^*2*^ = 0.823, *P* < 0.001), TDS (*r*^*2*^ = 0.367, *P* = 0.012) and Fe (*r*^*2*^ = 0.186, *P* = 0.031). Other environmental parameters involved in metabolite profile and bacterial composition discrimination on the second axis were Cr (*r*^*2*^ = 0.465, *P* = 0.006), Cu (*r*^*2*^ = 0.197, *P* = 0.042), and Zn (*r*^*2*^ = 0.131, *P* = 0.039). Lower pH, higher TDS and HM concentrations was positively associated with significant accumulation of AMP, inosine, methionine, carnitine and dimethylglycine with concomitant increase in the abundance of proteobacterial taxa (mainly γ- and β-proteobacteria) and Acidobacterial OTUs (Fig. 4c). However, these factors were negatively associated with abundance of members of phyla Firmicutes and Actinobacteria OTUs and uridine, dopa, asymmetric dimethylglycine, adenosine and phenylalanine accumulation.

### Effect of AMD Pollution on Rhizospheric Bacteria Catabolic Activity

**Fig. 5:**
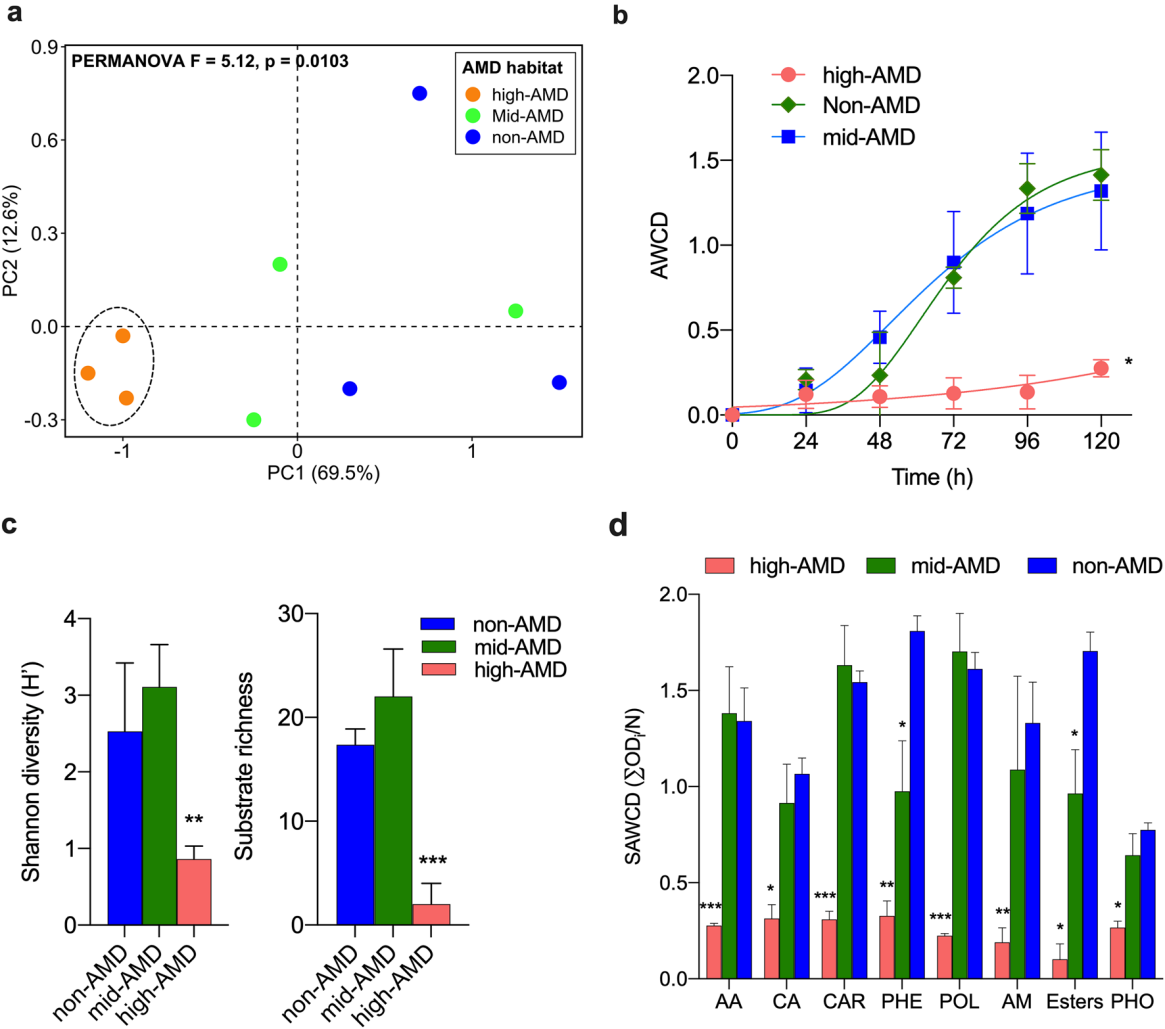
Community-level patterns of rhizosphere microbial carbon metabolism in *P*. *australis* under different AMD pollution. **a** PCA plot showing patterns of carbon utilization. The dashed line indicates significant cluster based on PERMANOVA (*P* < 0.05). **b** Average well color development (AWCD) at 96 h of incubation. **c** Shannon diversity index and substrate richness based on CLPP profile at 96 h of incubation. **d** Substrate average well color development (SAWDC) index at 96 h of incubation for different carbon substrate guilds. *AA* amino acids, *CA* carboxylic acids, *CAR* carbohydrates, *PHE* phenolic compounds, *POL* polymers, *AM* amines, esters; and *PHO* phosphorylated chemicals. ***, **, and *Indicate significant differences based on Tukey’s test at *P* < 0.001, *P* < 0.01, and *P* < 0.05

CLPP results revealed a clear distinction between carbon degradation profile of rhizospheric microbiota of *P*. *australis* under high AMD habitat and other sites (Fig. 5a, PERMANOVA *F* = 5.12, *P* = 0.0103). Further, both mid- and non-AMD rhizospheric communities also exhibited significant (*P* < 0.01) overall efficient utilization of carbon sources and higher metabolic activity and diversity than high-AMD soils based AWCD analysis, SR, Shannon diversity indices and SAWCD index (Fig. 5c).

Overall, high-AMD samples exhibited significantly (*P* < 0.05) very low efficiency in the utilization of all groups of carbon sources. In contrast, 6 Amino acids, 10 carbohydrates and 4 polymers were efficiently utilized by both mid- and non-AMD rhizospheric communities. However, a significant lower utilization of phenolics (2-hydroxy benzoic acid, 4-hydroxy benzoic acid) and esters (pyruvic acid methyl ester) was observed for mid-AMD samples. Collectively, these results suggest that the rhizosphere microbial community under low AMD pollution are metabolically more versatile than under high AMD pollution.

## Discussion

### Rhizosphere and Endosphere Metabolic Profile Differ Across AMD Pollution Gradient

Under metalliferous environment, plant species secrete a cocktail of small to high molecular organic and inorganic molecules in the rhizosphere. These secretions not only create a nutrient-rich microenvironment that may select for specialized metal-tolerant microbial communities [6], but may also participate directly or indirectly in HM metal detoxification process [13]. In this study, differential perturbation of primary metabolome profiles was observed between *P*. *australis* rhizosphere and endosphere compartments. Interestingly, only the rhizospheric microenvironment exhibited spatially distinct metabolome according to AMD pollution gradient (Fig. 1a). Specifically, 18 metabolites ranging from compatible osmolytes such as sugars, amino acids, organic acids, vitamins and pyridine nucleotides showed differential spatial exudation by root localization (rhizosphere vs. endosphere). Similarly, several studies have reported higher accumulation of sugars and sugar alcohols [12], amino acids and its derivatives [23], vitamins [12, 24] and pyridine nucleotides [12]. These molecules act as important protective responses to abiotic stress by terrestrial and aquatic plants. Huang et al. [25] also reported that two aquatic macrophytes *Z*. *latifolia* and *P*. *australis* diffuse oxygen and primary and secondary metabolites into rhizosphere. The modified root environment plays an important role in the selection and proliferation of symbiotic microbial communities. Toyama et al. [26] also reported that *P*. *australis* can release up to 70 mg of dissolved organic carbon per g root wet weight per day, which represents a large carbon resource for rhizosphere bacteria.

Under conditions of metal toxicity, plants reprogram metabolic processes related to plant growth and development, inducing higher levels of amino acids, its derivatives and organic acids [14]. In this study, ALDEx2 analysis revealed significant elevation in the abundance of ophthalmic acid, guanosine, niacinamide, histamine, methionine, thymidine and choline in the rhizosphere (Fig. 2a, c). In contrast, adenosine, symmetric dimethylglycine, isoleucine, guanosine monophosphate, dopa, adenine, asymmetric dimethylglycine, tyrosine and cytidine were enriched in the endosphere (Fig. 2). These results reflect storage of different metabolites in dissimilar tissues and cell types depending on their functionalities. Amino acids in plants may contribute to the detoxification process by regulating ion transport, chelating ions, and N metabolism under HM stress [12]. Association between elevated methionine and niacinamide and the signaling, sequestration and transportation of HMs such as Fe, Cu, Zn, and Cd has previously been reported [12]. Ophthalmic acid or γ-glutamyl-l-2-aminobutyryl-glycine (a tripeptide analogue of GSH) is a common oxidative stress marker in both plants and animals [27]. In contrast, quaternary ammonium compounds (choline and *N*,*N*-dimethylglycine) have been linked to the synthesis of membrane phospholipids and neurotransmitters key to various biological functions in plants [28]. Pyrimidine metabolite (thymidine) have also been implicated in signaling pathways linked to progressive drought-stress tolerance in bread wheat [29]. Elevated ophthalmic acid, guanosine, niacinamide, histamine, methionine, thymidine and choline (Fig. 2), therefore, suggest their likely importance in the root-sediment interface of *P*. *australis* under AMD pollution.

Clear differences in the rhizosphere exudates between the sites was also observed (Fig. 1), providing evidence that metabolite profiles are shaped by AMD habitat. Based on ALDEx2 analysis (Fig. 2b, d), differential abundance of several metabolites was observed between non-AMD and AMD sites. AMP, inosine, methionine, carnitine, and dimethylglycine were overrepresented in both mid and high-AMD sites. In support of these results, NMDS plot also revealed significant higher correlation between lower pH, higher TDS and HM concentrations (Cu, Cr, Fe, and Zn) and rhizospheric abundance of the aforementioned metabolites (Fig. 4c). In general, aquatic macrophytes have the ability to influence the surrounding water pH by their roots through either release of OH^−^ or HCO_3_^−^ or CO_3_^2−^, release of organic anions and inactivation of H^+^-ATPase, to increase pH [9]. A link between increase in pH due to organic acids exudation and Cr stress in rice rhizosphere has been previously reported [9]. In two wetland species, *Eriophorum angustifolium* and *Eriophorum scheuchzeri*, root-mediated pH changes has been associated with the bioavailability of Cd, Cu, Pb, and Zn from AMD and mine tailings [30]. On the other hand, rhizospheric overproduction of ammonium compounds such as methionine, carnitine and dimethylglycine may represent a major driver for root nutrient foraging strategies under low fertility AMD soils. The resultant plant ammonium nutrition may promote sugars exudation [31]. Furthermore, increased carnitine exudation may imply its role in promoting the fidelity of *P*. *australis* growth and cell division under AMD environment by imparting the antioxidative and protective effect against the genotoxic effect of HM stress [32]. These results indicate that under increasing AMD pollution different compartments of *P*. *australis* roots exude differential primary metabolites key to growth and root fidelity towards HM stress and detoxification.

### Rhizosphere and Endospheric Bacterial Community Structure Differ by AMD Pollution Gradient

Previous reports have demonstrated that the microbial community structure of root-associated microbiomes is affected by the plant compartment [33]. Consistent with these results, species richness and diversity indices of bacterial community for rhizosphere and endosphere of *P*. *australis* differed significantly in this study. However, dependence on sampling sites, with these indices decreasing with increasing AMD pollution was observed (Table 1). Surprisingly, lower bacterial diversity was observed for the rhizosphere than endosphere samples. We have previously reported a strikingly higher fungal diversity in the endosphere than rhizosphere compartments [3]. In contrast, several studies have reported reduced microbial diversity nearer to roots, with further reduction in the endosphere [4, 6]. However, the impact of pH and HM pollution was observed for rhizophere than endosphere. These results may imply that local *P*. *australis* root microenvironment has a complex effect on both bacterial and fungal community, selecting for more endophytic metal tolerant groups under AMD-stress conditions.

In general, the rhizosphere provides an environment for plant–microbes interaction due to the richness of nutrients necessary for the growth of both plants and microbes. Overall, OTUs assigned to phylum Firmicutes, Proteobacteria, Actinobacteria, Planctomycetes, Acidobacteria and Bacteriodetes (Fig. 3a) were the abundant *P*. *australis* root communities. Members of Proteobacteria (Alphaproteobacteria, Gammaproteobacteria), Actinobacteria, and Bacteroidetes have been reported to be dominant and ubiquitous in the rhizosphere of *P*. *australis* under diverse habitats [2, 7] and other terrestrial and aquatic plants [37]. As r-strategy organisms, Alphaproteobacteria and Gammaproteobacteria are capable of utilizing a broad range of root-derived carbon substrates [25]. This may explain, in part, their high abundances in the rhizospheric compartment. The high abundance of Actinobacteria, whose members are known to produce a wide variety of antibiotics, could contribute to pathogen resistance. On the other hand, enrichment of Bacteriodetes may indicate their importance in decomposing high molecular weight organic matter [38].

The phylum Firmicute*s* was dominant in both rhizosphere and endosphere of *P*. *australis* in non-AMD site. At lower taxonomic levels, the high abundance of potentially obligate anaerobes belonging to genus *Clostridium* and *Exiguobacterium* in non-AMD site rhizosphere and endosphere relate to the regulation of oxygen. In support of current findings, Huang et al. [25], reported that members of Firmicutes were enriched in *Z*. *latifolia* and *P*. *australis* rhizosphere sediments and bulk under freshwater ecosystem. In freshwater sediment bacterial communities, it is hypothesized that oxygen availability is a major determinant of community composition. Hernández et al. [37] also reported high abundance of members of genus *Clostridium*, an obligate anaerobes possessing strictly anaerobic fermentative metabolism, in anaerobic habitat of rice fields. In contrast, *Exiguobacterium* is a cosmopolitan bacterial genus that includes many extremophiles capable of surviving in both marine and nonmarine environments worldwide, including plant rhizosphere and freshwater sediments [39]. OTUs belonging to *Rhizobium*, *Delftia*, *Bradyrhizobium* and *Mesorhizobium* accounted for the major genera in rhizosphere and root endosphere of *P*. *australis* under AMD pollution. Both nitrogen-fixing rhizobial symbionts [8, 40] and diazotrophic *Delftia* [41] are known plant growth promoting (PGP) microbes, and HMs, organic and inorganic bioremediators in the rhizosphere. HM tolerant rhizobial strains have been reported from nutrient-poor contaminated sites to effectively carry out symbiotic nitrogen fixation, while legume–rhizobia symbiosis is widely known to detoxify HM and improves the quality of contaminated soils [40]. On the other hand, studies have shown that *Delftia* strains can resist HMs such as Zn, Hg, Pb, and Cr(VI) [42] and can transform HM into less toxic compounds [41, 43].

The two AMD environment rhizosphere samples also showed the abundance of bacteria in the order Bacillales (genus *Exiguobacterium*), Burkholderiales (genus *Massilia*), Actinomycetales (genus *Arthrobacter*) and Planctomycetales (unclassified *Planctomyceteae* PAC000036). *Arthrobacter* species has been reported to resist and bioaccumulate metals such as Cd, Co, Zn, Cr, and Hg due to their ability to accumulate chromate reductase (*ChrR*, *YieF*, *NemA* and *LpDH*) and NADPH-specific mercuric reductases (*MerA*) in the cytoplasm [44]. Similarly, tolerance of *Bacillus* and *Massilia* strains to Cd stress has previously been attributed to either binding of Cd^2+^ as cadmium phosphate and simultaneous increase in K^+^ uptake in the presence of Cd^2+^ ions [45]. One unique observation of this study was higher abundance of genus *Methylocystis* in the endosphere samples from high AMD polluted environment (Fig. 3c). As methanotrophs, *Methylocystis* strains have high ability to oxidize the greenhouse gas methane accounting to high denitrification activity in eutrophic waters similar to AMD wetland [46]. Further, Shi et al. [47] has also reported that at least eight genera of methanotrophs including *Methylocystis* possess both Hg(II) and As(V) reductases key to their tolerance to the two toxic HM and their bioremediation in polluted water. Therefore, aforementioned microbes colonizing the rhizosphere and endosphere gives clues to their potential roles in the nutrient uptake to promote the growth and HM stress tolerance of the *P*. *australis* under AMD environment.

Multivariate analyses showed clustering of both bacterial community based on AMD pollution, root compartments (endosphere *vs.* rhizospere) and associated root exudation (Fig. 4c). Specifically, lower pH, higher TDS and HM concentrations (Cu, Cr, Fe, and Zn) contributed significantly to lower bacterial diversity, particularly under heavy AMD pollution load. However, pH exhibited a significant stronger positive correlation with change in bacterial structure and diversity than other environmental factors (Fig. 4c). These results are consistent with other studies that indicate pH constitute a key determinant of bacterial community structure in different habitats [7, 34]. Mendez-Garcia et al. [35] also reported the strong correlation between the TDS, conductivity, Fe and other HM, and the microbial structure, and biomass in polluted AMD soils. Aguinaga et al. [36] also reported differential bacterial community structure and diversity dependent on suspended metal concentrations, sediment metal concentrations and other water chemistry parameters (pH and conductivity), and plant presence under wetland environment. This imply that increasing HM pollution and lower reduces microbial diversity and richness, and metabolic activity structure of the *P*. *australis* rhizosphere under AMD pollution [36]. Interestingly, lower pH, higher TDS and HM concentrations (Cu, Cr, Fe, and Zn) also strongly contributed to spatial perturbation of metabolites such as AMP, inosine, methionine, carnitine, and dimethylglycine with concomitant increase in the abundance of γ- and β-proteobacteria and Acidobacterial OTUs (Fig. 4c). In contrast, negative correlation was observed between these factors, Firmicutes and Actinobacterial OTUs as well as uridine, dopa, asymmetric dimethylglycine, adenosine, and phenylalanine accumulation. In summary, the results of present study give a snapshot on how *P*. *australis* utilize root exudation to improve its fitness in AMD-polluted wetland ecosystem to modulate the surrounding rhizospheric soil physical and chemical conditions to enhance nutrient uptake, and elicit symbiotic microbiome assembly responses.

### Catabolic Activity of Bacterial Community Exhibited AMD Pollution-Dependent Gradient

Complementing the metagenomic analyses, CLPP indices such as AWCD and SAWCD showed significant differences in the metabolic potential/carbon utilization capacity of the rhizospheric microbiota of *P*. *australis* according to AMD gradient (Fig. 5a). In addition, SR and Shannon diversity indices (H′), showed that both mid-AMD and non-AMD had higher metabolic activities than the high AMD site (Fig. 5b, c). Comparatively, mid-AMD site exhibited higher but not significant SR and H′, indicating that mild AMD pollution had appreciable effect in modulating the bacterial community richness and carbon degradation profile. In support of these findings, Chaerun et al. [48] observed increased activity, biomass, and functional diversity of the microbial communities in polluted soils compared unpolluted with elevated salinity, sodocity, and HM soils. Thus, the major changes in the chemical and physical properties of wetland due to AMD pollution have lasting impacts on the microbial communities of *P*. *australis* rhizosphere. These results suggests that rhizospheric effect maybe a key factor in the recruitment of symbiotic microbiome. The concomitant rhizospheric enrichment contributes to plant fidelity and the HM bioremediation under AMD polluted environment.

Overall, indices related to the structure of microbial catabolic activity under high-AMD pollution showed discernible decrease indicative of stress response (Fig. 5). Similar to our findings, Xiao et al. [49] has reported that HM (Cd and Pb) pollution negatively impacts on microbial growth, diversity and enzyme activity in soil. It suffices to speculate that extreme HM pollution towards the upper limit for cellular life significantly reduces *P*. *australis* rhizospheric bacterial community size and functional activity. This supports the hypothesis that the masking of rhizospheric effect under the high metal conditions become the bigger constraint in shaping the bacterial community structure and metabolic diversity.

## Conclusion

Our study characterized the unexplored link between bacterial community structure and metabolic diversity, and root exudation of *P*. *australis* under AMD-polluted environment. Overall, AMD pollution significantly affected the root primary metabolomic profile, the impact being more pronounced in the rhizosphere than endosphere. Elevated abundance of ophthalmic acid, guanosine, niacinamide, histamine, methionine, thymidine and choline imply their importance in the root-sediment interface of *P*. *australis* under AMD pollution. In the rhizosphere, elevation of AMP, inosine, methionine, carnitine, and dimethylglycine, and their correlation with pH, TDS and HM content (Cu, Cr, Fe, and Zn) under mid and high-AMD sites provides clues on their potential contribution towards *P*. *australis* growth, and root fidelity related to HM stress response and detoxification. In addition, novel strategies that promote *P*. *australis* growth, and root fidelity under AMD habitat based on rhizosphere microecology and root metabolomics analyses were highlighted. Under AMD pollution, *P*. *australis* not only remold their root metabolite exudation profile vital for the recruitment of beneficial rhizosphere bacteria (e.g., *Bacillus*, *Rhizobium*, *Delftia*, *Bradyrhizobium* and *Mesorhizobium)* for promotion of plant growth under pH and HM stress, but also induce some microbial taxa (e.g., *Delftia*, *Massilia*, *Arthrobacter*, *Bacillus*, and *Methylocystis*) key to HM detoxification processes. This study, therefore, provides insights on the understanding of novel auxiliary strategies utilised by *P*. *australis* to confer HM adaptability, sequestration and bioremediation capacity. A theoretical basis for further application and enhancing *P*. *australis* phytorhizoremediation efficiency has also been provided. However, this study focused only on the primary root exudates (metabolites) that were all assumed to be of plant origin. This may limit the overall understanding of the complex and multifaceted plant–microbe–metal interactions. Hence, a more comprehensive picture on the specific roles of root- and microbiome-specific metabolites in promoting *P*. *australis* growth and health under AMD-polluted environment need to be further explored incorporating the metatranscriptomic, metaproteomic, ionomics and other omics technologies. In addition, the aforementioned microbial taxa also need to be identified clearly and researched further.

## Supplementary Information

Below is the link to the electronic supplementary material.

Supplementary file1 (XLSX 11 KB)

Supplementary file2 (XLSX 20 KB)

Supplementary file3 (XLSX 12 KB)

Supplementary file4 (XLSX 19 KB)

## Data Availability

Data Associated with this study has been deposited at the NCBI database (https://www.ncbi.nlm.nih.com) as BioProject ID PRJNA742387. All other data generated are included within the article and supplementary materials.
